# Supplementary figures and images for: Biochemical reconstitution of heat-induced mutational processes
Source: PLoS One. 2024 Sep 17;19(9):e0310601. doi: 10.1371/journal.pone.0310601 (PMC11407675; doi:10.1371/journal.pone.0310601)

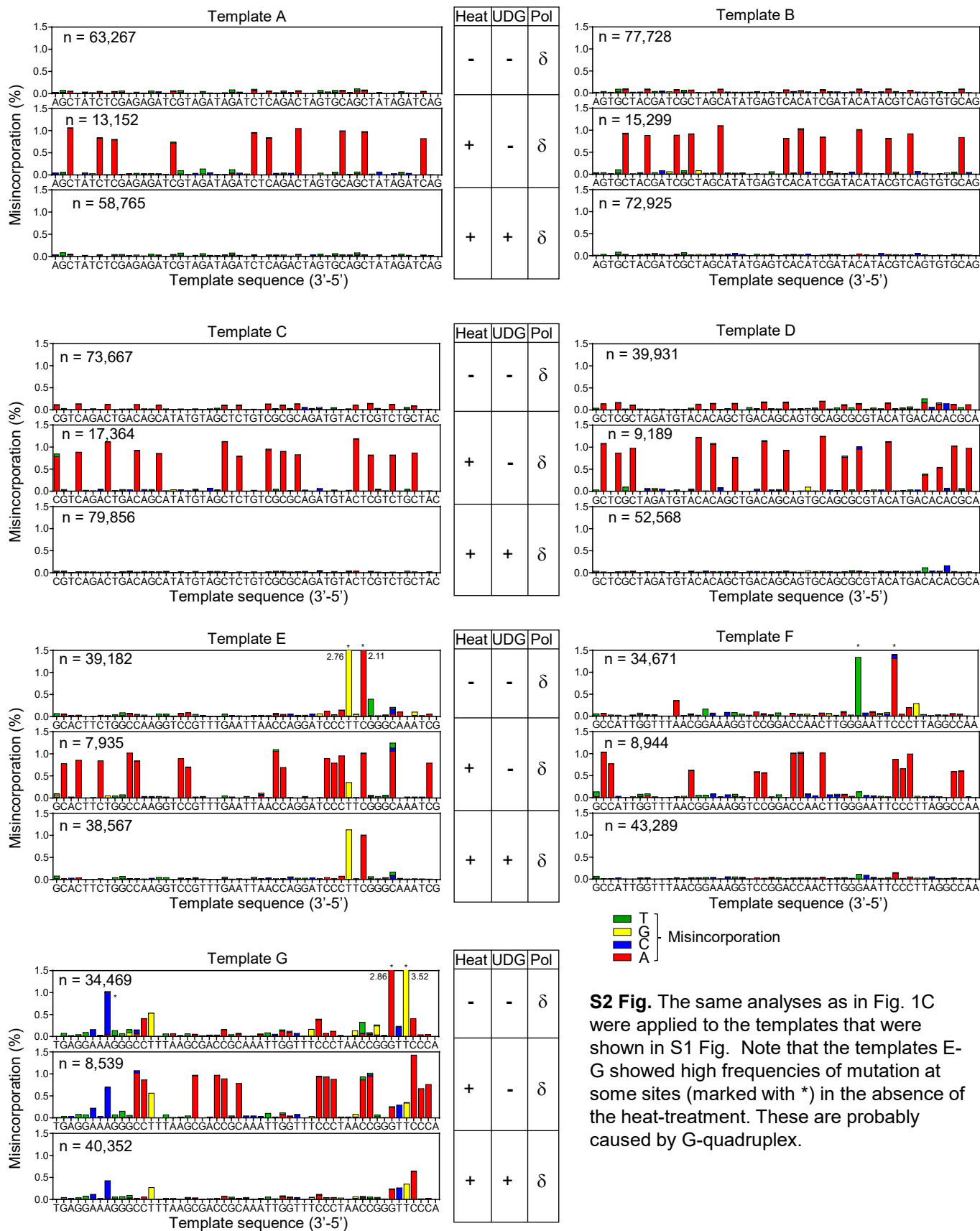

Supplement: S2 Fig — (PDF) [file pone.0310601.s002.pdf]
