# Supplementary material for: Biochemical reconstitution of heat-induced mutational processes
Source: PLoS One. 2024 Sep 17;19(9):e0310601. doi: 10.1371/journal.pone.0310601 (PMC11407675; doi:10.1371/journal.pone.0310601)
Supplement: S3 Fig — (PDF) [file pone.0310601.s003.pdf]

SIG	1-Cos
SBS1N	0.948956112
SBS2N	0.271035761
SBS3N	0.182293771
SBS4N	0.059045929
SBS5N	0.754802281
SBS6N	0.894633146
SBS7aN	0.405468718
SBS7bN	0.472845221
SBS7cN	0.188633991
SBS7dN	0.056080815
SBS8N	0.137234039
SBS9N	0.438840494
SBS10aN	0.142014911
SBS10bN	0.489609732
SBS10cN	0.596034793
SBS10dN	0.090068218
SBS11N	0.093896106
SBS12N	0.016417927
SBS13N	0.030242213
SBS14N	0.419166762
SBS15N	0.599932471
SBS16N	0.128680571
SBS17aN	0.030085769
SBS17bN	0.011465956
SBS18N	0.583337318
SBS19N	0.223686007
SBS20N	0.475831543
SBS21N	0.12861131
SBS22N	0.025844365
SBS23N	0.285738032
SBS24N	0.185657435
SBS25N	0.811267254
SBS26N	0.255886035
SBS27N	0.190125103
SBS28N	0.169624013
SBS29N	0.541728277
SBS30N	0.492775645
SBS31N	0.320158701
SBS32N	0.617521548
SBS33N	0.209996022
SBS34N	0.221248496
SBS35N	0.094551549
SBS36N	0.255143694
SBS37N	0.336499054
SBS38N	0.111884181
SBS39N	0.351359217
SBS40N	0.136107501
SBS41N	0.567722285
SBS42N	0.077778875
SBS43N	0.016917281
SBS44N	0.554989446
SBS45N	0.070713279
SBS46N	0.283908456
SBS47N	0.121204702
SBS48N	0.00222965
SBS49N	0.006221689
SBS50N	0.2940885
SBS51N	0.474827409
SBS52N	0.039972522
SBS53N	0.182705509
SBS54N	0.391910362
SBS55N	0.18475978
SBS56N	0.105079214

S3 Fig. Cosine similarity between the in vitro mutation spectrum (shown in Fig 2H) and normalized cancer mutational signatures (ver 3.2).
