# Supplementary material for: Biochemical reconstitution of heat-induced mutational processes
Source: PLoS One. 2024 Sep 17;19(9):e0310601. doi: 10.1371/journal.pone.0310601 (PMC11407675; doi:10.1371/journal.pone.0310601)
Supplement: S4 Fig — (PDF) [file pone.0310601.s004.pdf]

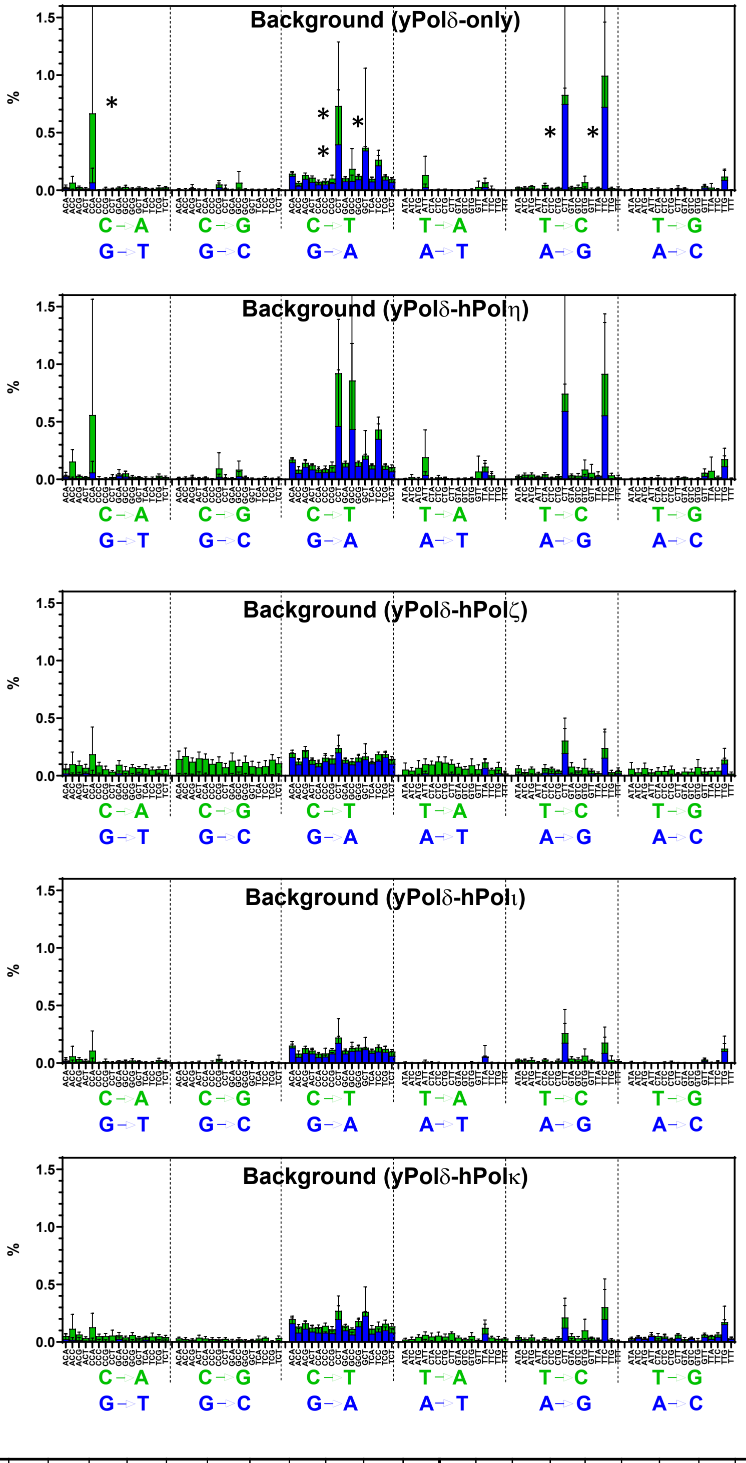

	ACA	ACC	ACG	ACT	CCA	CCC	CCG	CCT	GCA	GCC	GCG	GCT	TCA	TCC	TCG	TCT	ATA	ATC	ATG	ATT	CTA	CTC	CTG	CTT	GTA	GTC	GTG	GTT	TTA	TTC	TTG	TTT
Green	5	4	5	6	5	4	4	5	6	4	7	4	5	6	9	9	4	8	6	4	9	4	7	4	6	11	5	4	4	4	4	4
Blue	9	5	10	5	4	4	4	6	4	4	4	8	5	4	9	5	6	8	5	4	8	5	7	5	5	5	5	4	4	5	4	4

S4 Fig. Background spectra that were produced by indicated DNA polymerases. All Figures in the main body of the paper, except for Fig. 1C, present the data after subtracting the background. Note that background G>A that were observed consistently (~0.1%) should be due to U residues in the original synthetic DNA templates. Also note that the high background bars (marked with * in the top panel) are caused by the potential G-quadruplexes as shown in S2 Fig. Table shows the numbers of triplets in the templates. These numbers are considered 'n' values to calculate average mutation frequency and SD of each triplet.
