# Supplementary material for: Biochemical reconstitution of heat-induced mutational processes
Source: PLoS One. 2024 Sep 17;19(9):e0310601. doi: 10.1371/journal.pone.0310601 (PMC11407675; doi:10.1371/journal.pone.0310601)
Supplement: S1 Fig — (PDF) [file pone.0310601.s001.pdf]

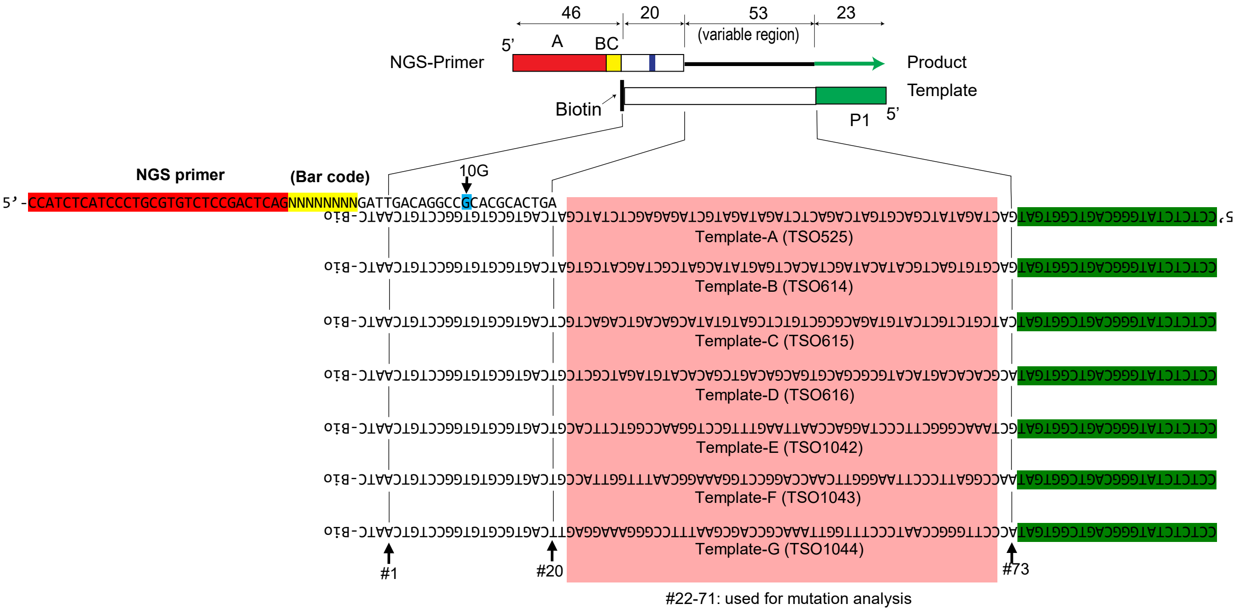

S1 Fig Sequences of synthetic ssDNA templates (template A-G) and a NGS primer.
All sequences were published previously (Sugiyama, et al., 2019).
